# Phase I dose escalation pharmacokinetic assessment of intravenous humanized anti-MUC1 antibody AS1402 in patients with advanced breast cancer

**DOI:** 10.1186/bcr2409

**Published:** 2009-10-07

**Authors:** Mark D Pegram, Virginia F Borges, Nuhad Ibrahim, Jyotsna Fuloria, Charles Shapiro, Susan Perez, Karen Wang, Franziska Schaedli Stark, Nigel Courtenay Luck

**Affiliations:** 1 Division of Hematology/Oncology, University of Miami Sylvester Comprehensive Cancer Center, University of Miami Miller School of Medicine, 1475 NW 12th Avenue; Suite 3400 (D8-4), Miami, FL 33136, USA; 2 Division of Medical Oncology, University of Colorado Denver, 12801 E 17th Avenue, MS 8117, Aurora, CO, USA; 3 Department of Breast Medical Oncology, UT MD Anderson Cancer Center, 1155 Pressler Street, CPB5.3540, Houston, TX, 77030, USA; 4 Department of Hematology/Oncology, Ochsner Clinic Foundation, 1514 Jefferson Hwy, New Orleans, LA 70121, USA; 5 Division of Medical Oncology, James Cancer Center, B405 Starling Loving 320 West 10th Avenue, Columbus, OH 43210, USA; 6 Hoffmann La Roche Pharmaceuticals, 340 Kingsland Street, Nutley, NJ 07110, USA; 7 Hoffmann La Roche Pharmaceuticals, Konzern-Hauptsitz, Grenzacherstrasse 124, CH-4070 Basel, Switzerland; 8 Antisoma Research Ltd, Chiswick Park Building 5, 566 Chiswick High Road, London, W4 5YF, UK

## Abstract

**Introduction:**

MUC1 is a cell-surface glycoprotein that establishes a molecular barrier at the epithelial surface and engages in morphogenetic signal transduction. Alterations in MUC1 glycosylation accompany the development of cancer and influence cellular growth, differentiation, transformation, adhesion, invasion, and immune surveillance. A 20-amino-acid tandem repeat that forms the core protein of MUC1 is overexpressed and aberrantly glycosylated in the majority of epithelial tumors. AS1402 (formerly R1550) is a humanized IgG1k monoclonal antibody that binds to PDTR sequences within this tandem repeat that are not exposed in normal cells. AS1402 is a potent inducer of antibody-dependent cellular cytotoxicity (ADCC), specifically against MUC1-expressing tumor cells. The objective of this study was to determine the safety, tolerability, and pharmacokinetic (PK) characteristics of AS1402 monotherapy in patients with locally advanced or metastatic MUC1-positive breast cancer that had progressed after anthracyclines- and taxane-based therapy.

**Methods:**

Patients received AS1402 over a 1- to 3-hour intravenous (i.v.) infusion at doses between 1 and 16 mg/kg, with repeated dosing every 1 to 3 weeks (based on patient-individualized PK assessment) until disease progression. Serum AS1402 levels were measured at multiple times after i.v. administration. Human anti-human antibody (HAHA) responses were measured to determine the immunogenicity of AS1402. Noncompartmental pharmacokinetic parameters were determined and were used to assess dose dependency across the dose range studied.

**Results:**

Twenty-six patients were treated. AS1402 was generally well tolerated. Two grade 3/4 drug-related adverse events were reported, both at the 3-mg/kg dose. Neither was observed in expanded or subsequent dosing cohorts. No anti-human antibodies were detected. Plasma concentrations of AS1402 appeared to be proportional to dose within the 1- to 16-mg/kg dose range assessed, with a mean terminal half-life of 115.4 ± 37.1 hours.

**Conclusions:**

Repeated iv administration of AS1402 was well tolerated, with a maximum tolerated dose (MTD) exceeding 16 mg/kg, the highest dose administered in this study. The half-life and exposure of AS1402 were such that weekly dosing could achieve plasma concentrations corresponding to the maximal ADCC activity observed *in vitro*. A phase II study is ongoing to evaluate the clinical activity of AS1402 in patients with advanced breast cancer.

**Trial registration:**

ClinicalTrials.gov Identifier: NCT00096057.

## Introduction

MUC1 is a cell-surface glycoprotein that establishes a molecular barrier at the epithelial surface and engages in morphogenetic signal transduction. Alterations in MUC1 glycosylation accompany the development of cancer and influence cellular growth, differentiation, transformation, adhesion, invasion, and immune surveillance [1]. MUC1 is overexpressed in more than 90% of breast cancers and the majority of epithelial tumors and has prognostic value in a number of malignancies, including breast cancer [2]. MUC1 is expressed as a stable heterodimer composed of two subunits derived from a single polypeptide chain, after cleavage in the endoplasmic reticulum [3]. The MUC1 N-terminal subunit contains a variable number of 20-amino-acid tandem repeats that are modified by *O*-linked glycans. The MUC1 C-terminal subunit comprises a 58-amino-acid extracellular domain, a 28-amino-acid transmembrane domain, and a 72-amino-acid cytoplasmic tail. This C-terminal domain accumulates in the cytosol of transformed cells and is delivered to the nucleus and mitochondria [4]. The MUC1 cytoplasmic domain associates with β-catenin and with the p53 tumor suppressor and is subject to phosphorylation by the epidermal growth factor receptor, c-Src, and glycogen synthase kinase-3β, suggesting a role for MUC1 in the erbB receptor kinase and Wnt signaling pathways [5].

MUC1 is often highly overexpressed in breast cancer relative to normal breast epithelial cells. Recently, Wei *et al*. [6] demonstrated that the C-terminal fragment of MUC1 associates with the DNA-binding domain of the estrogen receptor. Such binding stabilized the estrogen receptor by reducing ubiquitination and proteasomal degradation of the estrogen receptor. MUC1 also increased recruitment of coactivators SRC1 and GRIP1 and was associated with increased ER-α-mediated transcription. Taken together, these data suggest a role for MUC1 oncoprotein in estrogen-mediated cell growth and survival of breast cancer cells.

Antibodies targeting MUC1 epitopes studied in human breast tumor biopsies bind to at least 90% of invasive breast neoplasms [7]. The overexpression of MUC1 correlates with adhesion and invasion of breast cancer cells *in vitro *[8]. Breast cancer patients who demonstrate MUC1 overexpression in greater than 75% of tumor cells and aberrant subcellular localization (cytoplasmic and membranous) have significantly poorer disease-free and overall survival [9]. AS1402 (formerly R1550) is a humanized IgG1κ monoclonal antibody (huHMFG1, [10]) which binds (*K*_d_~1 nmol/L) the extracellular MUC1 peptide sequence, PDTR. These sequences are not exposed in normal cells because of full glycosylation, but aberrant glycosylation in cancer cells exposes the epitope to the antibody. AS1402 is a potent inducer of antibody-dependent cellular cytotoxicity (ADCC), specifically against MUC1-expressing tumor cells.

Snijdewint *et al*. [11] demonstrated ADCC elicited by AS1402 *in vitro *in the breast tumor cell line ZR-75-1, in three bone marrow-derived tumor cell lines from breast cancer patients (KM22, 1590, HG15), and in Chinese hamster ovary (CHO) cells transfected with the human *MUC1 *gene, by using peripheral blood mononuclear cells (PBMCs) from healthy donors. Cell lines that did not express human MUC1 were not susceptible to ADCC in the presence of AS1402. Very weak or no killing of the MUC1-positive target cell line with human PBMCs was noted in the absence of AS1402. These authors also demonstrated a strong reduction in specific AS1402-dependent cell killing of ZR-75-1 cells when the PBMCs were depleted of CD56^+^cells (that is, natural killer (NK) cells). Partial to complete depletion (> 50% to 90%) of either CD4^+^, CD8^+^, or CD19^+^cells (that is, T and B cells) from the PBMCs did not significantly reduce the ADCC. Moreover, the ADCC activity of AS1402 was shown to be dependent on the involvement of the Fcγ III receptor (CD16) on NK cells.

Immunotherapy for the treatment of cancer is an attractive alternative to cytotoxic chemotherapy. Since the approval in 1997 of rituximab (anti-CD20) for the therapy of non-Hodgkin lymphoma, several other antibodies have been licensed for different cancers. Many of these antibodies act by inhibiting signal transduction, and others, such as rituximab, trastuzumab (anti-HER2), and alemtuzumab (anti-CD52), act in addition through ADCC. NK cells of the body's immune system are directed to destroy antibody-targeted tumor cells. It has been reported that a natural humoral immune response to MUC1 protein in early breast cancer patients results in improved disease-free survival [12]. Interestingly, those patients with endogenous anti-MUC1 antibodies had a significantly higher probability of freedom from distant metastases, raising the possibility that the antibodies may be destroying circulating MUC1-positive tumor cells.

The objectives of this multicenter phase I study were to determine the safety and pharmacokinetics as well as the maximal tolerated dose (MTD) of AS1402 in patients with metastatic breast cancer.

## Materials and methods

### Patient eligibility

Patients with advanced or metastatic breast cancer were eligible for this clinical trial. Before initiation of the study at each investigational site, relevant study documentation was submitted to and approved by the responsible local ethics committee: Colorado Multiple Institutional Review Board, Aurora, CO; Ochsner Clinical Foundation Institutional Review Board, New Orleans, LA; The University of Texas, MD Anderson Cancer Center Surveillance Committee, Houston, TX; Office for the Protection of Research Subjects, Los Angeles, CA; and Western Institutional Review Board, Olympia, WA.

The guidelines of the World Medical Association Declaration of Helsinki in its revised edition (Edinburgh, Scotland, October 2000), the guidelines of ICH GCP (CPMP/ICH/135/95), as well as the demands of national drug and data-protection laws and other applicable regulatory requirements were strictly followed. Written informed consent was obtained from each patient before any study-specific screening procedures were undertaken.

### Inclusion criteria

Patients had to have histologically or cytologically confirmed breast cancer with overexpression of the MUC1 antigen on central immunohistochemistry assessment. Subjects had locally advanced or metastatic disease and had to have received no more than three prior chemotherapy regimens (including adjuvant/neoadjuvant therapy). They had to have previously received, with unsuccessful results, an anthracycline and a taxane in any combination for the treatment of breast cancer, unless ineligible for these treatments owing to comorbidities or refusal of therapy. In addition, patients whose tumors were HER2 positive had to have relapsed after treatment with trastuzumab (Herceptin). No restriction was posed for prior hormonal or biologic therapies or both.

### Exclusion criteria

Concurrent cytotoxic chemotherapy for metastatic breast cancer was not allowed. Patients with a left ventricular ejection fraction of less than 45%, as determined by multigated acquisition scan (MUGA) or echocardiogram (ECHO) scans within 4 weeks of study entry, were excluded.

### Treatment plan

It was planned to test doses of 1 mg/kg, 3 mg/kg, 9 mg/kg, and 16 mg/kg, according to the toxicity and pharmacokinetic (PK) profile observed at prior dose levels. The dose of AS1402 would not be increased beyond 16 mg/kg, as this was considered to be the maximum viable dose. It was planned that 24 evaluable patients would be recruited into the study, with six patients allocated to each treatment group.

AS1402 was administered over a 60-minute period for the 1-mg/kg and 3-mg/kg cohorts. The infusion times were increased to 120 minutes and 180 minutes for the 9-mg/kg and 16-mg/kg cohorts, respectively. For patients in the 1-mg/kg and 3-mg/kg cohorts, the first two treatments were given 21 days apart. The half-life after the first dose was determined for each patient, and the dosing intervals for dose 3 onward were set on an individual patient basis to be within ± 3 days of the half-life, in multiples of 7 days. For patients in the 9-mg/kg and 16-mg/kg cohorts, the dosing interval in multiples of 7 days was determined for the whole cohort, based on PK analysis of the data from all previous cohorts. Patients remained on treatment until they had disease progression or dose-limiting toxicity (DLT).

Cohort expansion to nine patients occurred if DLT was observed in any cohort. Adverse events (AEs) were coded according to the National Cancer Institute (NCI) Common Terminology Criteria for Adverse Events (CTCAE), version 3.0. DLT was defined as nonhematologic and hematologic grade 3 or greater, grade 2 or greater allergic reaction (bronchospasm and generalized urticaria), or grade 2 or greater autoimmune reaction. The MTD was defined as the highest dose studied at which the incidence of DLTs was less than 33%.

In each cohort, a single patient was treated initially and observed for at least 21 days. If no DLT occurred in the first patient, then two additional patients were treated at the same dose level and observed for 21 days. If either none or one of the three patients experienced a DLT, then the cohort was expanded to six patients. If at any given dose level, the first patient experienced a DLT, then one more patient was enrolled at that dose level and observed for 21 days before accrual of additional patients.

### Analytic methods and pharmacokinetics

Blood samples for pharmacokinetic analysis were drawn before infusion, at the end of infusion, at 4, 6, and 12 h after the start of infusion, and on days 2, 3, 4, 5, 8, 11, and 15. Analysis of AS1402 in human serum samples used a two-step solid-phase enzyme-linked immunosorbant assay (ELISA) with bovine serum albumin conjugated to the 20-amino-acid peptide sequence recognized by AS1402 as the bound antigen [11]. The lower limit of detection of this assay was 0.5 μg/ml.

Noncompartmental pharmacokinetic parameters were calculated from individual patient serum concentration-time profiles by using WinNonlin v4.0 (Pharsight, Mountain View, CA, USA). The maximum serum concentration of AS1402 (C_max_) was obtained directly from observed data. The elimination half-life was estimated from the terminal phase of the serum concentration-time profile. The area under the serum concentration-time profile extrapolated to infinity (AUC_0-∞_) was calculated by using the log-linear trapezoidal rule. The total serum clearance (CL) and the volume of distribution at steady state (V_ss_) were calculated by using the standard software formulae of CL = Dose/AUC_0-∞ _and V_ss _= CL*MRT (where the mean residence time was derived from the area under the first moment versus time curve).

Levels of tumor markers (CA15.3, CEA, and CA27.29) were measured by using a chemiluminometric assay at time points to coincide with radiologic assessments.

## Results

### Patient demographics

Patient demographics are shown in Table 1. A total of 26 female patients were enrolled in four dosing cohorts. Twenty-three percent of patients with known HER-2 status (5 of 22) were HER2-positive, in line with published prevalence data [13]. All patients had progressive disease after chemotherapy, with trastuzumab therapy also having failed for the HER2-positive patients.

**Table 1:** Patient demographics

Female patients enrolled (*n*)	26
Age (years) (median/min/max)	55.5/32.0/72.0
Mean weight ± SD (kg) (range)	73.1 ± 14.6 (50-108)
Median months from diagnosis of metastatic disease to study entry (months) (range)	14.4 (1.6-67.2)
**WHO performance status (n = 26)**	
0	17
1	9
**Steroid hormone receptor status (n = 24)**	
ER and/or PR^+^	18
ER and PR^-^	8
**HER2 status^a ^(n = 22)**	
HER2^+^	5
HER2^-^	17
**Metastatic tumor sites (n = 26)**	
Lung	17
Bone	15
Liver	13
Lymph nodes	12
Lesions on more than one organ	22
**Race (n = 26)**	
Caucasian	20
Asian	2
Hispanic	1
African-American	3

^**a**^HER2 status confirmed by fluorescence in situ hybridization (FISH) and immunohistochemistry (IHC).

### Clinical safety and tolerability

All 26 enrolled patients were evaluable for safety. The 1-mg/kg cohort enrolled three patients; the 3-mg/kg cohort was expanded to nine patients owing to the occurrence of a DLT. Consequently, an additional observation period before accrual of additional patients was implemented; no additional DLTs were observed that prevented enrollment into the subsequent cohort. The 9- and 16-mg/kg cohorts enrolled six and eight patients, respectively. The median number of doses received per patient was three (range, three to four), four (one to 13), six (four to 21), and 6.5 (three to 15) in cohorts 1, 2, 3, and 4, respectively. Although an MTD was not reached, the dose of AS1402 was not escalated above 16 mg/kg, which was considered to be the maximum viable. No patients with positive titers for human anti-human antibody (HAHA) were found in any cohort.

A summary of the patient- and investigator-reported drug-related clinical AEs is shown in Table 2. AS1402 was generally well tolerated, with few clinically significant drug-related AEs reported. The most frequently reported AE was grade 1 or 2 study drug-related reactions, (26% grade 1, 17.4% grade 2), described as nausea, fatigue, pyrexia, and pain or discomfort at the i.v. infusion site. The next most frequent adverse event was gastrointestinal toxicity (grade 1) in the form of nausea, constipation, or stomatitis. Reversible elevations of hepatic-function tests also were observed in some patients (grade 1: 8.7%, grade 2: 8.7%, grade 3: 4.3%, and grade 4: 4.3%).

**Table 2:** Drug-related adverse events (n = 26 patients)

Category of AE	Grade 1	Grade 2	Grade 3	Grade 4
**General disorders** (fatigue, pain, pyrexia, catheter-site edema, injection site burning)	6 (26.0%)	4 (17.4%)	-	-
**GI disorders ** (aphthous stomatitis, constipation, nausea)	4 (17.4%)	-	-	-
**Nervous system disorders** (dysguesia)	2 (8.7%)	-	-	-
**Skin and subcutaneous tissue disorders** (rash, alopecia)	5 (19.2%)	-	-	-
**Laboratory investigations** (↑ ALT, ↑ AST, ↑ GGT, ↑ bilirubin)	-	1 (4.3%)	1 (4.3%)	1 (4.3%)
**Metabolism and nutritional disorders ** (anorexia, hyperglycemia)	2 (8.7%)	-	-	1 (4.3%)
**Hepatobiliary disorder** (jaundice)	-	-	1 (4.3%)	-

Grade 3/4 drug-related AEs were reported for two patients among the 26 dosed with AS1402, both in the 3-mg/kg cohort. One patient had elevated ALT (grade 3), elevated AST (grade 4), jaundice (grade 3), increased blood bilirubin (grade 3), and elevated γ-glutamyl transferase (grade 3) that were considered possibly drug related and constituted a DLT. These events were of 0 to 2 days' duration, with the exception of jaundice, which persisted from day 14 to the time of death at day 26 due to progressive disease involving liver and bone metastases. Another patient experienced grade 4 hyperglycemia that was possibly related to treatment. This resolved after 14 days after antiglycemic therapy and delay in administration of AS1402. No grade 3/4 AE was observed in expanded or subsequent dosing cohorts. No grade 5 AEs were found. One patient was discontinued from the study owing to metastases to the central nervous system, and one patient died during the study of metastatic breast cancer; neither was considered to be related to the study medication. Eight patients died after they had been discontinued from the study; six died of breast cancer, and one died because of collapse of the lung. For one patient, the cause of death was unknown.

### Pharmacokinetics

**Figure 1:**
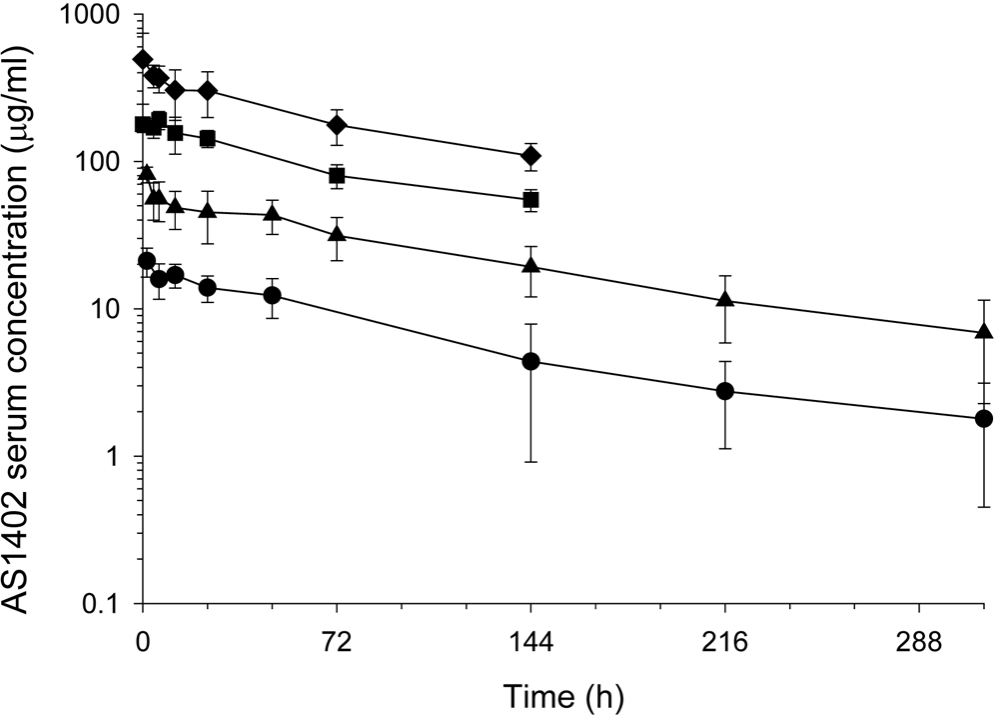
Mean AS1402 serum concentration-versus-time profiles (± SD) after i.v. administration to patients at doses of 1 mg/kg (circle), 3 mg/kg (triangle), 9 mg/kg (square), or 16 mg/kg (diamond). An antibody concentration of 10 μg/ml was sufficient to elicit ADCC to kill breast cancer cells *in vitro*.

The mean serum concentrations of AS1402 after the first dose for the 1-, 3-, 9-, and 16-mg/kg treatment groups are shown in Figure 1. A multiexponential decline in serum concentrations of AS1402 was observed, and systemic exposure increased with each successive dose escalation. Steady-state serum concentrations did not appear to have been reached in the majority of the patients during study treatment. The mean terminal half-life of AS1402 appeared shorter at the higher dose levels (111 and 102 h at the higher two doses, compared with 147 and 128 h at the lower two doses). Because the sampling schedule was shorter for the higher-dose cohorts, the shorter half-life values are likely to represent an earlier phase of the serum concentration-time curve, potentially including a partial distribution phase along with a terminal elimination phase. Although the initial doses were given 21 days apart, the PK data supported a reduction of the dosing interval to 7 days.

**Figure 2:**
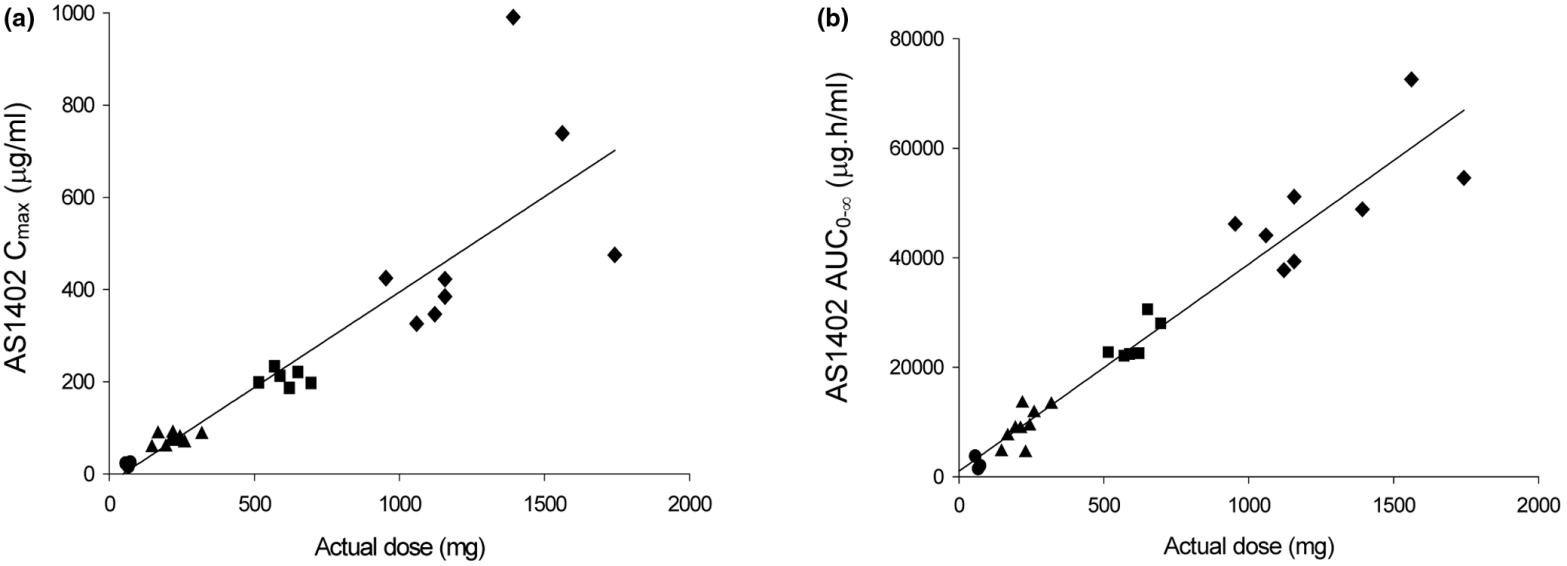
AS1402 C_max _**(a)** and AS1402 AUC_0-∞ _**(b)** versus dose proportionality in patients receiving doses of 1 mg/kg (circle), 3 mg/kg (triangle), 9 mg/kg (square), or 16 mg/kg (triangle).

The calculated noncompartmental pharmacokinetic parameters are summarized in Table 3. Clearance of AS1402 from serum was consistent across all dose cohorts, with mean values ranging from 0.34 to 0.49 ml/h/kg. The volume of distribution was broadly comparable across the treatment cohorts, with mean values ranging from 50.3 to 68.2 ml/kg. AS1402 exhibited linear pharmacokinetics with respect to dose across the 1- to 16-mg/kg dose range, as demonstrated by the dose-proportional increase in C_max _and AUC_0-∞ _(Figure 2a and 2b, respectively).

**Table 3:** Noncompartmental pharmacokinetic parameters for AS1402 after i.v. administration (mean ± SD)

Dose cohort	1 mg/kg (n = 3)	3 mg/kg (n = 9)	9 mg/kg (n = 6)	16 mg/kg (n = 8)
C_max_ (μg/ml)	21.1 ± 4.67	73.5 ± 11.8	209 ± 17.3	514 ± 232
Half-life (h)	147 ± 19.3	128 ± 50.6	111 ± 23.0	102 ± 22.6
AUC_0-∞_(μg*h/ml)	2,399 ± 1,205	9,032 ± 3,314	24,757 ± 3,625	49,302 ± 10,971
CL (ml/h/kg)	0.486 ± 0.213	0.386 ± 0.174	0.369 ± 0.0488	0.337 ± 0.0648
V_ss _(ml/kg)	68.2 ± 16.9	54.6 ± 12.2	56.8 ± 7.14	50.3 ± 18.56

### Tumor markers

Sixteen (62%) patients had elevated CA15.3 levels at screening and throughout the study. CEA levels remained relatively unaltered by treatment with AS1402, and no clear trends in correlation between CA27.29 and exposure to AS1402 were found.

### Clinical activity

Twenty-two patients were evaluable for efficacy. Objective clinical response was assessed according to RECIST. No objective complete or partial responses were recorded during dose escalation; five (22.7%) patients had a best overall response of stable disease (one in the 3-mg/kg cohort, one in the 9-mg/kg cohort, and three in the 16-mg/kg cohort); stable disease durations ranged from 80 to 119 days. All these patients had progressive disease before antibody therapy. The median time to tumor progression by cohort is summarized in Table 4.

**Table 4:** Time to tumor progression (TTP) by dose cohort

Dose cohort	1 mg/kg (n = 3)	3 mg/kg (n = 6)	9 mg/kg (n = 6)	16 mg/kg (n = 7)
Median TTP (days)	39	39	40	44
25^th^-75^th ^percentile	33-46	38-80	31-41	36-80
Range	33-46	35-117	28-119	35-106

## Discussion

These are the first data from a clinical study of a MUC1-targeting naked antibody. In total, 26 evaluable patients were recruited into the study; doses of 1 mg/kg, 3 mg/kg, 9 mg/kg, and 16 mg/kg were tested. The 16-mg/kg dose was considered to be the maximum viable dose. Repeated i.v. administration of AS1402 was well tolerated, with an MTD exceeding 16 mg/kg. The majority of drug-related AEs were NCI CTC grade 1 or 2. Infusion-associated reactions were generally CTC grade 1. No incidents of patients with positive titers for anti-human antibodies were found during the study.

Systemic exposure of AS1402 appears to be linear with respect to dose within the 1- to 16-mg/kg dose range assessed. This linear relation between exposure and dose was suggestive that the pharmacokinetic disposition of this humanized anti-MUC1 antibody is not dependent on saturable distribution or clearance processes, which might be expected if significant binding to circulating antigen occurred *in vivo*. Previous *in vitro *studies have shown that the affinity of AS1402 for its antigen in solution is 100-fold lower than that for cell-bound antigen, which provides a good rationale for the lack of binding to circulating antigen *in vivo *[14]. The mean terminal half-life of AS1402 was measured to be approximately 5 days, which suggests that weekly dosing of AS1402 at doses greater than 3 mg/kg would be required to achieve serum antibody concentrations at or above the 10 μg/ml observed for maximal ADCC activity *in vitro*. A 5-day half-life for AS1402, a humanized antibody (approximately 90% human), is comparable with the published values of 4.7 days for cetuximab (chimeric anti-EGFR, approximately 66% human) and 7.5 days for panitumumab (fully human anti-EGFR) [15]. Although the initial proposed dosing interval was 21 days, a weekly administration would be an acceptable addition to standard treatment regimens.

The best overall response was stable disease (achieved by five patients), and the median time to disease progression ranged from 39 to 44 days. Patients enrolled into this study had been heavily pretreated and had progressive disease on entry. In light of this, the fact that stable disease was observed in five patients warrants further investigation in less heavily pretreated or chemotherapy-naïve patients.

Antibodies have, in general, been most successfully applied as elements of combination regimens. An antibody against MUC1 has the attraction in this context that its target is overexpressed in around 90% of human breast cancers. Moreover, some data suggest a rationale for combination with existing breast cancer therapies, especially anti-estrogens. The MUC1 C-terminal subunit interacts with ERα, and this interaction is stimulated by 17β-estradiol (E2). Direct binding of MUC1 to the ERα DNA-binding domain stabilizes ERα by blocking its ubiquitination and degradation. Furthermore, MUC1 stimulated ERα-mediated transcription and contributed to the E2-mediated growth and survival of breast cancer cells [6].

Reports from a phase III trial of a breast cancer vaccine, Theratope, indicated that aromatase inhibitors may increase ADCC, adding to the rationale for combining anti-MUC1 antibodies with this approach to estrogen reduction. In a study reported by Braun *et al*. [16], tumor cells incubated with the aromatase inhibitor, formestane, became sensitized to killing by monocyte-mediated, antibody-dependent cellular cytotoxicity by an anti-MUC1 antibody. These observations led the authors to conclude that a hormone-based treatment may collaborate with antigen-specific tumor immunity to produce improved tumor control in patients with breast cancer. Results from the phase III study showed that patients receiving Theratope plus concomitant hormone therapy had a prolonged survival over those patients receiving a control vaccine plus hormone therapy [17]. Survival in these patients was also positively associated with immunoglobulin G titers to the underglycosylated mucin-associated glycoprotein, an antigen similar to that recognized by AS1402. A phase III trial combining trastuzumab with the aromatase inhibitor anastrazole found that patients treated with the combination therapy had a significantly higher response rate and progression-free survival than did patients receiving anastrazole alone [18].

## Conclusions

Data obtained from the AS1402 phase I clinical trial, together with the role of the MUC1 oncoprotein in stabilization and activation of the estrogen receptor and the potential for aromatase inhibitors to increase ADCC, provide a clear basis for a phase II study combining an anti-MUC1 antibody with endocrine therapy. Letrozole has been shown, in three separate trials, to be superior to anastrazole in reducing circulating estrogen levels [19]. A phase II randomized, open-label, multicenter study of weekly infusions of 9 mg/kg AS1402 in combination with letrozole as first-line treatment in postmenopausal women with locally advanced or metastatic breast cancer is ongoing.

## Abbreviations

ADCC: antibody-dependent cellular cytotoxicity; AE: adverse event; ALT: alanine transaminase; AST: aspartate transaminase; AUC: area under the curve; CHO: Chinese hamster ovary; CL: clearance; CTCAE: Common Terminology Criteria for Adverse Events; DLT: dose-limiting toxicity; ECHO: echocardiogram; ELISA: enzyme-linked immunosorbant assay; HAHA: human anti-human antibody; huHMFG: humanized human milk-fat globule; ICH GCP: International Conference on Harmonisation of Good Clinical Practice; i.v.: intravenous; MTD: maximum tolerated dose; MUGA: multigated acquisition scan; NCI: National Cancer Institute; NK: natural killer; PBMC: peripheral blood mononuclear cell; PK: pharmacokinetic.

## Competing interests

ESP and KW were employees of Hoffmann La Roche Pharmaceuticals; NCL was an employee of Antisoma Research Limited. The authors declare that they have no competing interests.

## Authors' contributions

MP, VB, NI, JF, and CLS contributed patients to the study and the acquisition of data; ESP, KW, FSS, and NCL contributed to the study design and data analysis. All the authors were involved in drafting the manuscript and have given final approval of the version to be published.
